# Association between High-Sensitivity C-Reactive Protein and Blood Pressure Variability in Subacute Stage of Ischemic Stroke

**DOI:** 10.3390/brainsci13070998

**Published:** 2023-06-28

**Authors:** Chuanli Xu, Zhiyong Fu, Wei Wu, Jin Zhang, Meitong Liu, Lianbo Gao

**Affiliations:** 1 Department of Neurology, The Fourth Affiliated Hospital of China Medical University, Shenyang 110032, China; 2 Department of Neurology, Xuanwu Hospital of Capital Medical University, Beijing 100053, China; 3 Department of Epidemiology, School of Public Health, China Medical University, Shenyang 110122, China

**Keywords:** cerebrovascular diseases, ischemic stroke, blood pressure variability, high-sensitivity C-reactive protein

## Abstract

The determinants of blood pressure variability (BPV) are complex. We aimed to evaluate whether circulating high-sensitivity C-reactive protein (hsCRP) is associated with short-term BPV during the subacute stage of ischemic stroke. In this observational study, a consecutive series of acute ischemic stroke patients who underwent 24 h ambulator blood pressure monitoring (ABPM) during day 4 to 10 after onset were enrolled. Multivariable linear regression models were constructed to assess relationships between hsCRP and BPV. Among a total of 325 patients analyzed, the mean age was 60 years old and 72% were male. The SD, CV, ARV of 24 h SBP and DBP were more likely to be higher in patients with hsCRP ≥ 2 mg/L, and these predispositions remained unchanged in linear regression analyses after adjusting for possible confounding factors, with a dose-response relationship when patients were additionally categorized into quartiles according to hsCRP levels using the lowest quartile as a reference category. In contrast, similar results were observed for the mean of SBP but not the mean of DBP. These results indicate that hsCRP is dose-dependently associated with short-term BPV during the subacute stage of ischemic stroke. These findings suggested that patients with a higher level of hsCRP tended to have larger blood pressure fluctuations.

## 1. Introduction

A hypertensive response occurs within 24 h in up to 80% of patients with acute ischemic stroke (AIS), and then blood pressure (BP) tends to decline spontaneously in two-thirds of patients over the subsequent few days [1]. It is well known that increased BP in the acute phase of ischemic stroke may affect stroke outcomes, but the optimal management of BP remains an issue of long-lasting debate [2,3]. BPV during the subacute stage, which may provoke or exacerbate secondary brain injury, has increasingly been recognized as a novel prognostic factor independent of mean BP levels [4,5,6,7], and may represent a new modifiable therapeutic target [8].

Mechanisms and determinants of very-short-term (beat-to-beat), short-term (for 24 h), mid-term (day-to-day), and long-term BPV (visit-to-visit) are probably different [9]. BPV in the short term is under delicate neural and humoral regulations, in which the arterial baroreflex plays a key role [10]. Baroreceptors are mechanosensitive neurons with peripheral projections in the vessel wall of the aortic arch and carotid sinuses that are activated by the vascular stretch induced by BP fluctuations [11]. The process of mechanoelectrical transduction in the baroreceptors depends on two components: the mechanical component determined by the viscoelastic properties, and the functional component modulated by various neurohumoral substances and paracrine factors, including inflammatory cells and other compounds [12,13].

Notably, ischemic stroke has a profound impact on systemic immunity, and abnormally enhanced systemic inflammation can perpetuate for months [14,15]. We hypothesized that activated systemic inflammatory response may increase short-term BPV. However, the association between circulating inflammatory biomarker hsCRP and BPV in subacute stroke patients has never been studied. Thus, we aimed to determine whether circulating hsCRP is associated with the variability of 24 h ABPM measurements during the subacute stage of ischemic stroke.

## 2. Methods

### 2.1. Standard Protocol Approvals, Registrations, and Patient Consents

This was a single-center cross-sectional study. All the procedures complied with the Declaration of Helsinki. The study protocol has been approved by the Ethical Committee of The Fourth Affiliated Hospital of China Medical University and each participant provided informed consent.

### 2.2. Patients

Patients admitted to the stroke unit in The Fourth Affiliated Hospital of China Medical University from 1 October 2019 to 30 September 2022 were consecutively enrolled. Patients were eligible if they were diagnosed as AIS with confirmation of computed tomography or magnetic resonance; underwent 24 h ABPM during the subacute phase of stroke; and consented to participate. The subacute stage of ischemic stroke was considered as the time period during which patients were neurologically stabilized. Operationally, it was defined as the time period after 4–10 days from symptom onset [7,16]. We excluded patients who had missing data and subjects with diseases that might substantially affect their levels of hsCRP, including poor kidney function, acute infection or a history of chronic infections, and previous malignancy carcinoma.

### 2.3. Data Collection

#### 2.3.1. General Assessment

Demographic characteristics and medical histories were collected at the time of enrollment. Patients were considered as smoking if they actively smoked within the last 12 months. Body mass index (BMI) was calculated as weight (kg) divided by the square of height (m). Laboratory findings such as white blood cell (WBC) counts, neutrophil (NEUT) counts, monocyte (MONO) counts, lymphocyte (LYM) counts, low-density lipoprotein (LDL), and high-density lipoprotein (HDL) were gathered. Stroke severity was assessed at admission by stroke neurologists using the National Institutes of Health Stroke Scale (NIHSS). Ischemic stroke was classified into five subtypes by following the Trial of ORG 10172 in Acute Stroke Treatment (TOAST) criteria [17]. All patients underwent brain MRI with diffusion-weighted imaging (DWI) sequence within 7 days after admission. Infarct volume was measured using automated in-house software based on DWI [18]. We evaluated lesion distribution based on the involved vascular territory. They were divided into three lesion patterns: anterior circulation, posterior circulation, and anterior and posterior circulation. The evaluation of each subject was performed by a senior neurologist blinded to the biomarkers. The treatment during hospitalization was according to the AIS management guideline. The ongoing usage of antihypertensive medications when the ABPM was performed was also recorded.

#### 2.3.2. hsCRP Assessment

Blood samples were obtained from all patients within 24 h after hospitalization and extracted and transported to the central laboratory at −80 °C through the cold chain until tests were carried out centrally and blindly. The concentrations of hsCRP were measured by immunoturbidimetry on a Roche Cobas C701 analyzer.

#### 2.3.3. Short-Term BPV Assessment

ABPM was performed during the subacute phase of stroke using a Meditech ABPM-05 device (Meditech Ltd., Budapest, Hungary). The time interval from stroke onset to performing 24 h ABPM was recorded. We obtained BP readings at 30 min intervals during the daytime and at 60 min intervals during the nighttime, eliminating the transition period when BP changed rapidly. Daytime was defined as from 6:00 am to 10:00 pm, nighttime as from 10:00 pm to 5:00 am, and the transition period was from 5:00 am to 6:00 am. Subjects with recorded BP readings less than 70% of the expected measurements were not included in the final analysis. The weighted means of SBP and DBP in the 24 h were recorded. The following parameters of BPV were calculated for the 24 h SBP and DBP, consistent with previous studies [19,20]: standard deviation (SD, key parameter for blood pressure variability because it is commonly used), coefficient of variation (CV, SD/BP_mean_ × 100), average real variability (ARV, a transformation of SD uncorrelated with mean blood pressure). We chose SD, CV, and ARV based on the previous literature suggesting that multiple approaches to measuring BPV should be employed.

### 2.4. Statistical Analysis

Baseline characteristics were described and compared by dichotomies of hsCRP whose distribution highly skewed to right (skewness = 3.47). We dichotomized the participants according to hsCRP levels into ≥2 mg/L or <2 mg/L. This cutoff was chosen because it is a well-established clinical cutoff to assess cardiovascular risk, based on previous analyses [21]. Continuous variables that followed a normal distribution were presented as mean ± SD and tested by Student *t*-test analysis; those that were skewed distribution were reported as median (interquartile range [IQR]) and compared using the Mann–Whitney test. Categorical variables were presented as frequencies (%) and were tested using the Chi-square test.

Linear regression models were used to examine the independent relationship of hsCRP with the mean BP, SD, CV, and ARV, respectively. Important covariates for BPV were selected based on our prior knowledge, and were adjusted in multivariable analyses (including age, sex, BMI, smoking, hypertension, diabetes mellitus, atrial fibrillation, coronary heart disease, prior TIA or stroke, initial NIHSS, onset to ABPM, antihypertensive therapy, LDL, HDL, TOAST classification, and lesion patterns). To assess dose response, patients were additionally categorized into quartiles according to hsCRP levels using the lowest quartile as a reference category. The P for trend was tested by entering the median value of each category of hsCRP as a continuous variable in each model. Linear regression assumptions were not violated and a variance inflation factor of <2.5 was used to test for collinearity. The β value was estimated with 95% CI. Subgroup analyses were performed by repeating the regression analysis after stratification by stroke severity (major stroke group: NIHSS > 3, minor stroke group: NIHSS ≤ 3), and heterogeneity across subgroups was quantified by formal interaction tests. Supplementary analyses separately revealed the association between monocytes and BPV by using Pearson correlation analysis.

Our study followed the guidelines for reporting observational studies [22]. All analysis was conducted using R version 4.1.2, and a two-tailed *p* value below 0.05 was considered to indicate statistical significance.

## 3. Results

A total of 431 AIS patients who underwent 24 h ABPM during the subacute phase were included in the initial assessment. This was after the further exclusion of patients who declined to participate or had missing data (n = 31), poor kidney function (n = 10), acute infection or a history of chronic infections (n = 53), and previous malignancy carcinoma (n = 12). As a result, 325 subjects were eventually included in this analysis. Of the 325 patients analyzed, the mean [SD] age was 60.0 [11.5] years old, and 234 (72%) were male. The most common prior medical condition was hypertension (70.8% [n = 230]), followed by diabetes mellitus (34.8% [n = 113]) and prior stroke or TIA (21.8% [n = 71]). The median initial NIHSS score was 2 (interquartile rage 1–5), and the median time interval from stroke onset to perform ABPM was 7 (interquartile rage 6–9) days. Nearly one-half of the patients had large-artery atherosclerosis (49.8% (162)). Furthermore, 62.2% (n = 202) of patients had infarcts in the anterior circulation. Compared with participants with lower hsCRP, those with higher hsCRP were more likely to be serious at admission, and with larger infarct volume. In addition, the hsCRP ≥ 2 mg/L group tended to have a higher level of circulating monocytes, white blood cells, and neutrophils, while the level of lymphocytes showed no difference (Table 1).

**Figure 1:**
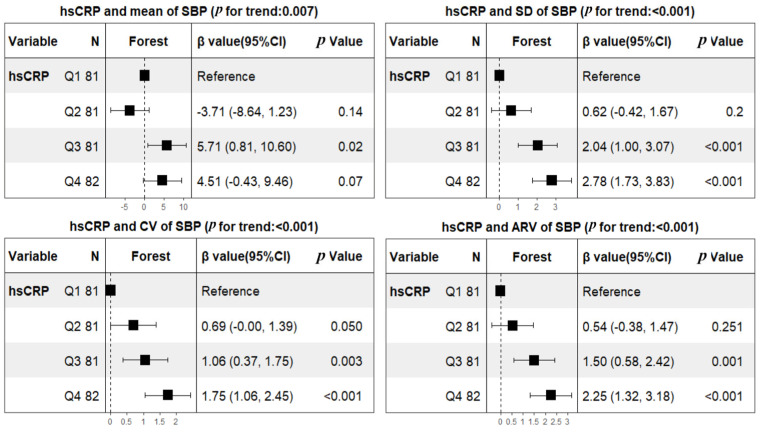
Multivariable analyses of associations between hsCRP quartiles and BPV of SBP during subacute stage of ischemic stroke Abbreviations: hsCRP = high-sensitivity C-reactive protein; SBP = systolic blood pressure; SD = standard deviation; CV = coefficient of variation; ARV = average real variability). Model adjustment was done for age, sex, BMI, smoking, hypertension, diabetes mellitus, atrial fibrillation, coronary heart disease, prior TIA or stroke, initial NIHSS, onset to ABPM, antihypertensive therapy, LDL, HDL, TOAST classification, and lesion patterns. hsCRP (mg/L) quartiles: Q1 [0.1, 0.64), Q2 [0.64, 1.57), Q3 [1.57, 3.45), Q4 [3.45, 33.52).

**Figure 2:**
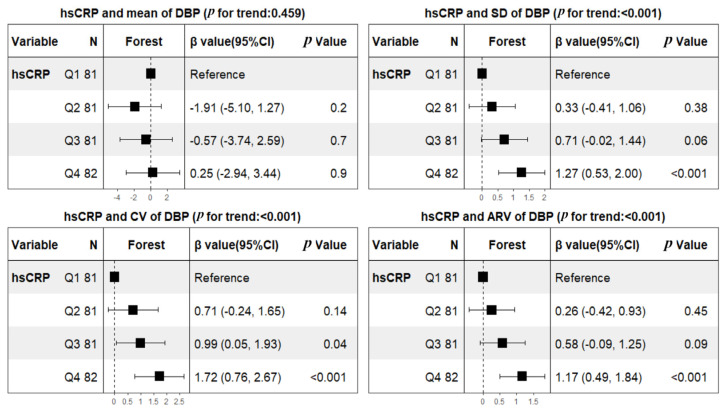
Multivariable analyses of associations between hsCRP quartiles and BPV of DBP during subacute stage of ischemic stroke Abbreviations: hsCRP = high-sensitivity C-reactive protein; DBP = diastolic blood pressure; SD = standard deviation; CV = coefficient of variation; ARV = average real variability. Model adjustment was done for age, sex, BMI, smoking, hypertension, diabetes mellitus, atrial fibrillation, coronary heart disease, prior TIA or stroke, initial NIHSS, onset to ABPM, antihypertensive therapy, LDL, HDL, TOAST classification, and lesion patterns. hsCRP (mg/L) quartiles: Q1 [0.1, 0.64), Q2 [0.64, 1.57), Q3 [1.57, 3.45), Q4 [3.45, 33.52).

The profiles of 24 h ABPM BP parameters in the subacute stage are summarized in Table 2. The weighted means of 24 h SBP and DBP were 142.1 ± 16.9 and 82.4 ± 10.9 mmHg, respectively. The mean values of SBP tended to be higher in patients with hsCRP ≥ 2 mg/L (147.2 ± 17.3 versus 138.2 ± 15.6, *p* < 0.001), but mean values of DBP did not (83.4 ± 12.5 versus 81.8 ± 9.5, *p* = 0.184). In contrast, both SBP and DBP fluctuations are larger in subjects with hsCRP at/above 2 mg/L. After adjusting for predetermined covariates of age, sex, BMI, smoking, hypertension, diabetes mellitus, atrial fibrillation, coronary heart disease, prior TIA or stroke, initial NIHSS, onset to ABPM, antihypertensive therapy, LDL, HDL, TOAST classification, and lesion patterns, linear regression analyses showed that hsCRP was not only significantly related to the BPV of SBP (β, 2.29 [95% CI 1.55–3.04] for SD; β, 1.21 [95% CI 0.72–1.71] for CV; β, 1.9 [95% CI 1.24–2.56] for ARV), but also the BPV of DBP (β, 0.93 [95% CI 0.41–1.46] for SD; β, 1.12 [95% CI 0.44–1.80] for CV; β, 0.93 [95% CI 0.45–1.41] for ARV). Not unexpectedly, hsCRP was significantly associated with mean SBP (β, 6.94 [95% CI 3.39–10.48]), but mean DBP was not (β, 0.99 [95% CI −1.29–3.27]) (Table 3). To further assess dose response, patients were additionally categorized into quartiles according to hsCRP levels using the lowest quartile as a reference category. Consequently, hsCRP was dose-dependently associated with mean SBP and all variability indices including SD, CV, ARV of SBP and DBP after adjusting for the same confounding factors mentioned above (Figure 1 and Figure 2).

Because minor stroke was predominant in our cohort, sensitivity analyses were performed by repeating the regression analysis in the subgroup of patients with minor stroke and the subgroup of patients with major stroke, respectively. After adjusting the same confounding factors above, identical results (all *p* for interaction > 0.05) were observed for SBP in the minor stroke subgroup (β, 2.49 [95% CI 1.45–3.53] for SD; β, 1.36 [95% CI 0.64–2.07] for CV; β, 2.19 [95% CI 1.24–3.14] for ARV) and major stroke subgroup (β, 2.18 [95% CI 1.00–3.35] for SD; β, 0.96 [95% CI 0.20–1.71] for CV; β, 1.83 [95% CI 0.82–2.85] for ARV) (Table 4), and similar patterns (all *p* for interaction > 0.05) were found for DBP in these two subgroups (minor stroke subgroup: β, 1.07 [95% CI 0.28–1.86] for SD; β, 1.41 [95% CI 0.39–2.43] for CV; β, 0.93 [95% CI 0.22–1.64] for ARV. major stroke subgroup: β, 0.82 [95% CI 0.03–1.61] for SD; β, 0.66 [95% CI −0.36–1.67] for CV; β, 0.80 [95% CI 0.07–1.52] for ARV) (Table 4). Supplementary analyses were conducted to explore associations between circulating monocytes and BPV. Pearson correlation analyses showed that monocytes were positively correlated with all variability indices of SBP (*r* = 0.177, *p* = 0.001 for SD; *r* = 0.165, *p* = 0.003 for CV; and *r* = 0.131, *p* = 0.020 for ARV); the associations of monocytes with the variability indices of DBP were similar (*r* = 0.184, *P* = 0.001 for SD; *r* = 0.159, *p* = 0.004 for CV; and *r* = 0.177, *p* = 0.001 for ARV), though the correlation coefficients were numerically smaller. Not surprisingly, neither mean SBP or DBP was related to monocytes (*r* = 0.042, *p* = 0.400 for SBP; *r* = 0.028, *p* = 0.600 for DBP).

## 4. Discussion

In this study, we found that hsCRP was dose-dependently associated with short-term BPV during the subacute stage of ischemic stroke, defined as the time period after 4–10 days from symptom onset. Concomitantly, similar results were observed for the mean of SBP but not the mean of DBP.

BP is a dynamic variable that experiences significant fluctuations after ischemic stroke. Numerous studies have suggested that BPV at various intervals during hospitalization is associated with hemorrhagic transformation [23], greater DWI lesion growth [24], early neurological deterioration [25], and increased risk of adverse clinical outcome at 3 months after ischemic stroke onset [26]. Generally, the impairment in autoregulation after stroke may leave the ischemic tissue susceptible to the fluctuations in flow generated by increased variability of BP [27]. An elevation in systemic BP exceeding the upper limit of mechanic autoregulation can lead to an increased risk of hemorrhagic transformation, whereas deviant decreases in BP can give rise to hypoperfusion or infarct extension [28]. Particularly, during the transitional period between the acute and chronic stages of ischemic stroke (the subacute stage), increased BPV may provoke secondary brain injury and exert a relatively prolonged detrimental influence on end-organ perfusion. Recent studies have highlighted subacute-phase BPV as a predictor of medium-and long-term clinical outcomes after ischemic stroke [4,5,6,7,16]. For example, Kang et al. [4] and Naito et al. [4] have separately reported that day-by-day BPV and 24 h ABPM BPV, but not average BP during the subacute phase, are associated with functional outcome at 3 months in AIS patients. In addition, Fukuda et al. found that the day-by-day variability of BP during the subacute stage of AIS was related to an increased long-term risk of recurrent stroke [7]. Furthermore, a randomized trial discovered an interesting phenomenon—that calcium-channel blockers reduce the risk of stroke more than expected on the same basis of mean BP alone, and that β blockers are less effective than expected [29]. A meta-analysis found that differences between their effectiveness can be explained by class effects on intraindividual variability in BP [30]. In addition, another study showed that the use of amlodipine (a calcium-channel blocker) to stabilize BPV led to a decrease in the incidence of coronary events [31]. Thus, a reduction in BPV during the subacute phase may improve long-term outcomes in patients with ischemic stroke.

AIS not only induces a local neuroinflammatory response but also has a profound impact on systemic immunity [15]. Inflammation plays a critical role in the pathogenesis and prognosis of ischemic stroke, and serum levels of inflammation biomarkers have been shown to persist at high levels in stroke patients at 3-month follow-up compared to baseline [32]. C-reactive protein, one of the most-investigated and well-acknowledged inflammatory markers, is reported to be positively associated with SBP within 24 h after stroke onset [33]. Our findings about the associations between hsCRP and the mean of SBP and DBP are consistent with these studies. However, whether hsCRP is related to BPV in subacute ischemic stroke patients remains unclear. Prior study showed that there are positive associations between CRP and BPV during 24 h ABPM in healthy adults, but the associations were of a fairly modest magnitude because healthy subjects had relatively normal inflammation and BP fluctuated within narrow ranges [34]. Our study firstly contributed strong evidence that hsCRP was dose-dependently related to intraindividual variability of 24 h BP in the subacute stage. Although there have been no such investigations in subacute stroke before, 24 h BP parameters in the present study were consistent with previous research that aimed to determine whether BPV in the subacute phase of ischemic stroke predicted clinical outcome [4]. Another key finding was that the variability indices of SBP and DBP tended to increase with the circulating level of monocytes in the subacute stroke, though the correlation coefficients were numerically smaller. Importantly, an animal study suggested that circulating lymphocyte numbers decline but levels of blood monocytes and neutrophils increase after brain ischemia, triggering a systemic immune response [14]. All these relationships provide clues that anti-inflammation as a modifiable strategy to maintain stable blood pressure may be possible. Particularly, with encouraging data in coronary heart disease, the efficacy and safety of anti-inflammatory agents in patients with AIS have been observed and are being further evaluated in RCTs (NCT02898610, NCT05439356) [35].

Short-term BPV is a complex phenomenon produced by multiple factors, including increased central sympathetic drive and a reduction in arterial reflex and humoral factors, among which the arterial baroreflex is most important [10]. How then, might inflammation influence 24 h BPV during the subacute stage of ischemic stroke? Biological mechanisms to explain this relationship were explored. First, as we assumed, we speculated that chronic systemic inflammation after stroke would disturb the baroreceptor mechanosensors (a functional component of baroreceptors related to ionic factors), leading to diminished baroreflex function and excess BPV. Physiologically, functional neural mechanisms, in addition to structural vascular changes, contribute importantly to altered baroreflex responses, and the changes in baroreceptor sensitivity mediated by functional components can occur independent of any change in vascular distensibility. Second, BPV may be a consequence of an autonomic imbalance, which can potentially lead to inflammatory response. Compelling evidence suggests that post-stroke immune responses can be modulated by the sympathetic nervous system [14]. Third, with the order reversed, increased BPV may also promote inflammation using biomechanical stimuli [36]. Explaining our results in terms of these mechanisms is necessarily speculative, and other explanations are possible.

### Study Strengths and Limitations

The strengths of our study include the high sensitivity of hsCRP and the uniform BP measurements of ABPM in a specified time period. There are also several limitations. Firstly, this is an observational study, and there might unavoidably be a selection bias. However, data collectors were blind to the probable results, suggesting that selection bias may be reduced. Secondly, the neurological deficits of all patients in our study were relatively minor (mean NIHSS at admission was 2 points), but further adjustment for NIHSS at admission did not affect the results, and analyses in subgroups of patients after stratifying by stroke severity showed comparable results. Thirdly, we did not take into account the levels of serum inflammatory markers over time. Blood samples were collected within 24 h of admission, but patients underwent 24 h ABPM in a time period from day 4 to 10 after onset, which may have introduced heterogeneity. However, studies have shown that enhanced systemic inflammation can perpetuate for months [15]. Finally, antihypertensive medications were empirically given in 28.3% of patients. Although our results were adjusted for the ongoing use of antihypertensive drugs, this may have affected the relationship between hsCRP and BPV.

## 5. Conclusions

Our study shows that hsCRP is dose-dependently associated with short-term BPV during the subacute phase of ischemic stroke, irrespective of age, sex, BMI, smoking, hypertension, diabetes mellitus, atrial fibrillation, coronary heart disease, prior TIA or stroke, initial NIHSS, onset to ABPM, antihypertensive therapy, LDL, HDL, TOAST classification, and lesion patterns. Further prospective studies are warranted to clarify the causal relationship between inflammation and short-term BPV. Then, anti-inflammatory therapy may be considered to reduce BP fluctuation.

## Figures and Tables

**Table 1:** Comparisons of baseline characteristics according to dichotomies of hsCRP.

Characteristics	Total (n = 325)	hsCRP < 2 mg/L (n = 186)	hsCRP ≥ 2 mg/L (n = 139)	*p* Value
Demographics				
Age, y, mean ± SD	60.0 ± 11.5	59.8 ± 10.5	60.1 ± 12.6	0.808
Male, n (%)	234 (72.0)	135 (72.6)	99 (71.2)	0.787
BMI, kg/m^2^, mean ± SD	26.1 ± 3.6	25.9 ± 3.5	26.3 ± 3.7	0.224
Smoking, n (%)	150 (46.2)	86 (46.2)	64 (46.0)	0.972
Medical histories, n (%)				
Hypertension	230 (70.8)	127 (68.3)	103 (74.1)	0.254
Diabetes mellitus	113 (34.8)	61 (32.8)	52 (37.4)	0.387
Atrial fibrillation	20 (6.2)	12 (6.5)	8 (5.8)	0.796
Coronary heart disease	24 (7.4)	11 (5.9)	13 (9.4)	0.241
Prior TIA or stroke	71 (21.8)	38 (20.4)	33 (23.7)	0.475
Clinical features				
Initial NIHSS, median (IQR)	2 (1, 5)	2 (1, 4)	3 (1, 6)	0.003
Infarct volume, mL, median (IQR)	1.9 (0.4, 22.8)	1.2 (0.3, 15.6)	2.5 (0.5, 34.8)	0.001
Onset to ABPM ^a^, d, median (IQR)	7 (6, 9)	8 (6, 9)	7 (6, 9)	0.122
Antihypertensive therapy ^b^, n (%)	92 (28.3)	51 (27.4)	41 (29.5)	0.681
WBC, ×10^^9^/L, mean ± SD	6.9 ± 1.8	6.4 ± 1.6	7.5 ± 1.8	<0.001
NEUT, ×10^^9^/L, mean ± SD	4.3 ± 1.5	3.9 ± 1.2	4.8 ± 1.5	<0.001
MONO, ×10^^9^/L, mean ± SD	0.5 ± 0.2	0.4 ± 0.1	0.5 ± 0.2	<0.001
LYM, ×10^^9^/L, mean ± SD	2.0 ± 0.7	1.9 ± 0.6	2 ± 0.7	0.089
LDL, mmol/L, mean ± SD	2.4 ± 0.8	2.3 ± 0.8	2.4 ± 0.8	0.459
HDL, mmol/L, mean ± SD	1.1 ± 0.3	1.1 ± 0.3	1.1 ± 0.4	0.870
TOAST classification, n (%)				0.216
LAA	162 (49.8)	85 (45.7)	77 (55.4)	
CE	18 (5.5)	12 (6.5)	6 (4.3)	
SVO	125 (38.5)	80 (43)	45 (32.4)	
OD	5 (1.5)	2 (1.1)	3 (2.2)	
UD	15 (4.6)	7 (3.8)	8 (5.8)	
Lesion patterns, n (%)				0.033
Anterior circulation	202 (62.2)	114 (61.3)	88 (63.3)	
Posterior circulation	92 (28.3)	60 (32.3)	32 (23)	
Anterior and posterior circulation	31 (9.5)	12 (6.5)	19 (13.7)	

Abbreviations: hsCRP = high-sensitivity C-reactive protein; BMI = body mass index; TIA = transient ischemic attack; NIHSS = National Institutes of Health Stroke Scale; IQR = interquartile range; ABPM = ambulator blood pressure monitoring; WBC = white blood cell; NEUT = neutrophil; MONO = monocyte; LYM = lymphocyte; LDL = low-density lipoprotein; HDL = high-density lipoprotein; LAA = large-artery atherosclerosis; CE = cardioembolism; SVO = small vessel occlusion; OD = other determined etiology; UD = undetermined etiology; ^a^ Time interval from stroke onset to perform ABPM. ^b^ Ongoing use of antihypertensive medications when the ABPM was performed.

**Table 2:** Comparisons of BP parameters according to dichotomies of hsCRP.

BP Parameters	Total (n = 325)	HsCRP < 2 mg/L (n = 186)	hsCRP ≥ 2 mg/L (n = 139)	*p* Value
SBP_mean_	142.1 ± 16.9	138.2 ± 15.6	147.2 ± 17.3	<0.001
SBP_SD_	13.8 ± 3.5	12.6 ± 3.0	15.2 ± 3.6	<0.001
SBP_CV_	9.7 ± 2.3	9.2 ± 1.9	10.5 ± 2.5	<0.001
SBP_ARV_	12.8 ± 3.1	11.9 ± 2.7	14 ± 3.1	<0.001
DBP_mean_	82.4 ± 10.9	81.8 ± 9.5	83.4 ± 12.5	0.184
DBP_SD_	9.9 ± 2.4	9.4 ± 2.3	10.5 ± 2.5	<0.001
DBP_CV_	12.1 ± 3.1	11.6 ± 2.8	12.8 ± 3.3	<0.001
DBP_ARV_	8.9 ± 2.2	8.4 ± 2.0	9.5 ± 2.4	<0.001

Abbreviations: hsCRP = high-sensitivity C-reactive protein; BP = blood pressure; SBP = systolic blood pressure; DBP = diastolic blood pressure; SD = standard deviation; CV = coefficient of variation; ARV = average real variability.

**Table 3:** Multivariable analyses of associations between dichotomous hsCRP and BPV during subacute stage of ischemic stroke ^a^.

Indices	Mean		SD		CV		ARV	
	β Value (95% CI)	*p* Value	β Value (95% CI)	*p* Value	β Value (95% CI)	*p* Value	β Value (95% CI)	*p* Value
SBP								
hsCRP ≥ 2 mg/L	6.94 (3.39, 10.48)	<0.001	2.29 (1.55, 3.04)	<0.001	1.21 (0.72, 1.71)	<0.001	1.9 (1.24, 2.56)	<0.001
DBP								
hsCRP ≥ 2 mg/L	0.99 (−1.29, 3.27)	0.392	0.93 (0.41, 1.46)	<0.001	1.12 (0.44, 1.8)	0.001	0.93 (0.45, 1.41)	<0.001

Abbreviations: hsCRP = high-sensitivity C-reactive protein; SBP = systolic blood pressure; DBP = diastolic blood pressure; SD = standard deviation; CV = coefficient of variation; ARV = average real variability. ^a^ Model adjustment was done for age, sex, BMI, smoking, hypertension, diabetes mellitus, atrial fibrillation, coronary heart disease, prior TIA or stroke, initial NIHSS, onset to ABPM, antihypertensive therapy, LDL, HDL, TOAST classification, and lesion patterns.

**Table 4:** Stroke severity stratified relationship between dichotomous hsCRP and BPV during subacute stage ^a^.

Subgroup	Mean		SD		CV		ARV	
	β Value (95%CI) *p* Value	P _interaction_	β Value (95%CI) *p* Value	P _interaction_	β Value (95%CI) *p* Value	P _interaction_	β Value (95%CI) *p* Value	P _interaction_
SBP								
Minor stroke ^b^	7.05 (1.76, 12.33) 0.009	0.491	2.49 (1.45, 3.53) <0.001	0.490	1.36 (0.64, 2.07) <0.001	0.277	2.19 (1.24, 3.14) <0.001	0.585
Major stroke ^b^	9.28 (3.96, 14.61) <0.001		2.18 (1.00, 3.35) <0.001		0.96 (0.20, 1.71) 0.013		1.83 (0.82, 2.85) <0.001	
DBP								
Minor stroke ^b^	0.66 (−2.53, 3.86) 0.682	0.554	1.07 (0.28, 1.86) 0.008	0.580	1.41 (0.39, 2.43) 0.007	0.302	0.93 (0.22, 1.64) 0.010	0.999
Major stroke ^b^	2.76 (−0.96, 6.48) 0.144		0.82 (0.03, 1.61) 0.043		0.66 (−0.36, 1.67) 0.202		0.80 (0.07, 1.52) 0.032	

Abbreviations: hsCRP = high-sensitivity C-reactive protein; SBP = systolic blood pressure; SD = standard deviation; CV = coefficient of variation; ARV = average real variability. ^a^ Model adjustment was done for age, sex, BMI, smoking, hypertension, diabetes mellitus, atrial fibrillation, coronary heart disease, prior TIA or stroke, initial NIHSS, onset to ABPM, antihypertensive therapy, LDL, HDL, TOAST classification, and lesion patterns. ^b^ Minor stroke refers to ischemic stroke with NIHSS score no more than 3, Major stroke refers to ischemic stroke with NIHSS score more than 3.

## Data Availability

The data used in this article will be shared by reasonable request from any qualified investigator.
